# Supplementary material for: Molecular Docking of Lactoferrin with Apoptosis-Related Proteins Insights into Its Anticancer Mechanism
Source: Int J Mol Sci. 2025 Feb 26;26(5):2023. doi: 10.3390/ijms26052023 (PMC11899785; doi:10.3390/ijms26052023)
Supplement: Supplementary file 1 [file ijms-26-02023-s001.zip › ijms-3482298-supplementary.pdf]

Supplementary Materials

Negative Control Docking Analysis

To assess the specificity of our docking results, we conducted a negative control docking experiment using Caspase 8 and Ovalbumin, a protein with no known interaction with apoptotic regulators. This test aimed to determine whether AlphaFold 3 could correctly distinguish between biologically relevant and non-specific interactions.

The docking metrics, summarized in Table S1, indicate a significantly lower iPTM score (0.12) and higher PAE values (27.57 Å–27.90 Å) for the negative control (Cas8-Ovalbumin), confirming the absence of meaningful interaction. In contrast, the hLf-Cas9 and hLf-Cas8 complexes exhibited higher iPTM scores (0.34 and 0.25, respectively), as shown in Figure S1A, supporting the greater stability of these interactions.

The PAE values for all complexes, displayed in Figure S1B, highlight that the negative control complex (Cas8-Ovalbumin) has the highest PAE Min and PAE Max values, reinforcing the lack of specific interaction. Meanwhile, the functional docking results exhibit lower PAE values, suggesting more reliable structural predictions.

Finally, the pTM scores, illustrated in Figure S1C, confirm that hLf-Cas9 and hLf-Cas8 have higher confidence values, while the negative control complex (Cas8-Ovalbumin) presents a significantly lower score, indicating low structural stability. These findings further support the robustness of our docking methodology, ensuring that observed interactions are biologically relevant rather than random artifacts.

Table S1. Summary of Docking Metrics

Complex	iPTM Score	PAE Min (Å)	PAE Max (Å)	pTM Score
hLf-Caspase-9	0.34	13.07	18.56	0.62
hLf-Caspase-8	0.25	14.57	14.64	0.58
Caspase-8-Ovalbumin	0.12	27.57	27.9	0.48

* Summary of docking scores for hLf-Cas9, hLf-Cas8, and the negative control (Cas8-Ovalbumin), including iPTM, pTM, and PAE values to assess interaction stability and confidence.

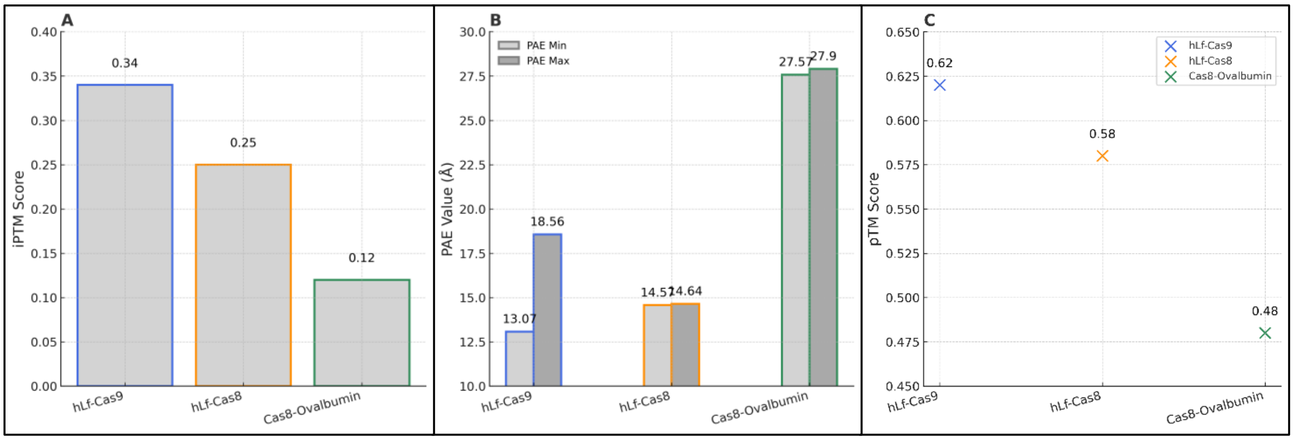

Figure S1. Docking Metrics for Functional and Negative Control Interactions. (A) iPTM Score Comparison: the higher iPTM scores for hLf-Cas9 and hLf-Cas8 suggest more stable interactions, while the negative control (Cas8-Ovalbumin) has a significantly lower score, indicating a lack of biologically

meaningful binding. **(B)** The Predicted Aligned Error (PAE) Comparison: Negative control shows significantly higher PAE Min and PAE Max values, reinforcing the absence of stable interaction. **(C)** pTM Score Comparison: the higher pTM scores for hLf-Cas9 and hLf-Cas8 indicate greater confidence in the docking predictions, whereas the negative control has a much lower pTM score, confirming its low structural stability.
